# Brain Enhancing Ingredients from Āyurvedic Medicine: Quintessential Example of *Bacopa monniera*, a Narrative Review

**DOI:** 10.3390/nu5020478

**Published:** 2013-02-06

**Authors:** Hemant K. Singh

**Affiliations:** Lumen Research Foundation, 1st Cross Street, 2nd Avenue, Ashok Nagar, Chennai-600083, India; E-Mail: hemant1943@gmail.com; Tel.: +91-522-2341160; Fax: +91-44-42033176

**Keywords:** *Āyurveda*, nutrition, geriatrics, rejuvenation, *Bacopa monniera*, memory, *Rasāyana*

## Abstract

*Āyurveda*, the science (*ved*) of life (*ayu*), owing its origin to Veda, the oldest recorded wisdom of human civilization written in 3500 BCE, contains extensive knowledge of various diseases and their therapeutic approaches. It essentially relied on nature and the immune system of an individual, and therapeutic interventions were introduced only to augment the immune system. *Āyurveda* had eight specialties, including psycho-neuroscience (a combination of psychology, clinical psychology and psychiatry) and a unique promotive therapy encompassing nutrition, rejuvenation and geriatrics. The symptoms of various brain disorders, including memory disorder, were well defined. The goal of *Āyurveda* was to help an individual to achieve his cherished goal of leading a healthy life of 100 years. To achieve this, great emphasis was laid on nutrition, diet and a good conduct by the two great exponents of *Āyurveda* viz. Carak and Suśruta. By following these regimens, an individual could lead a less stressful life free from emotional disturbances. Both Carak and Suśruta had believed that these in combination with *rasayana* (rejuvenating) plants could enable an individual to lead a healthy life of 100 years.

## 1. Introduction

*Āyurveda*, the science (*ved*) of life (*ayu*), is one of the most ancient medical systems of human civilization. It is considered to be an *upveda* (off-shoot) of Atharvaveda (the science of well-being) and, thus, has its origin from Veda, the oldest recorded wisdom of India written *c*. 3500 BCE. A science, based essentially on the fundamental laws of nature, *Āyurveda* has in its repository extensive knowledge and wisdom about almost each and every aspect of diseases and their therapeutic approach. The application of *Āyurveda* also extended to other economically and socially useful living beings. Thus, we also had *Hastāyurveda*, the treatise on treatment of elephants [1,2,3,4], *A śva-āyurveda*—a treatise on treatment of horses [5]—and *Vŗķşa-āyurveda*—the treatise dealing with the cultivation of trees and seasoning of woods to be used in ship building, *etc.* [6]. Almost all professions practiced in India till medieval time were based not only on physical strength and vigor, but also on mental prowess [7,8,9]. The game of hunting was greatly encouraged in the royal and warrior classes to revitalize and strengthen both the body and brain [10].

Vivid descriptions of trees, plants and other naturally occurring herbs are to be found in all the important treatises of India right from Veda. The epics **Ramayan** and **Mahabharat** describe a large numbers of medicinal plants. The magnum opus of the great medieval Indian bard Kalidās, viz. **Raghūvamśamahākāvyam,** is replete with vivid descriptions of plants beneficial for the treatment of diabetes. The treatise on state-craft, viz. **Arthśāstra,** written by Kāutilya (Chānakya) also gives a vivid description of medicinal plants and their use and, also, various diets suitable for various professions [11]. All this will give an idea about the importance attached to health and the all pervasive influence of *Āyurveda* on life and society in India. This review describes the main tenets and philosophy of *Āyurveda*.

### 1.1. What Is Āyurveda?

Before any mention of nutritive ingredients in *Āyurveda* is considered, it becomes imperative to understand certain fundamental tenets of *Āyurveda*. The mainstay of *Āyurveda* is two sets of original treatises consisting of three books each. These are: (a) *Vrhattraayi*, *i.e.*, three big books, viz. **Charaka Samhitā** (600 BCE), **Suśrutū Samhitā** (100 CE) and **Samhitā** of Vāgbhata (600 CE) and (b) *Laghūttrayi*, *i.e.*, three small books, viz. **Mādhav-Nidāna** (900 CE), **Sārangdhara Samhitā** (1300 CE) and **Bhāva Prakaśa Nighantū** (materia-medica of Bhāva Prakaśa written in 1500 CE). All these texts cover almost all the vital aspects of life, health, disease and their treatment by adopting a three pronged approach, viz. philosophical, holistic and also humanistic. *Āyurveda* has advocated a comprehensive composite health care system encompassing promotive, preventive and curative systems of medicine. It has been practiced since ancient times in the form of *Astānga Āyurveda* (literally meaning eight-fold *Āyurveda*) and has eight major clinical specialties, viz. *Kāyāchikitsā* (internal medicine), *Śālya Tantra* (Surgery), *Śālakya* (pertaining to the diseases of supra-clavicular regions, *i.e.*, diseases of the eyes, ear, nose and throat), *Kumārbhrtya* (pediatrics, including obstetrics and gynecology), *Bhūtvidyā* (literally meaning demonology, but actually psychology, including clinical psychology and psychiatry), *Agād Tantra* (toxicology), *rasāyan-Tantra* (promotive therapy, including nutrition, rejuvenation and geriatrics) and *Vājikaran* (sexology, pertaining to aphrodisiacs). 

### 1.2. Mental Ill-Health

**Figure 1:**
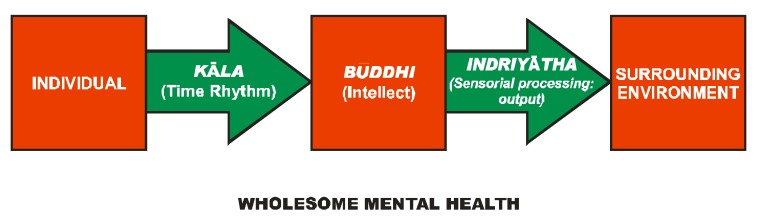
An individual reacts to their surrounding environment in a wholesome manner when all the three vital links of the axis, viz. *kāla* (time rhythm), *būddhi* (intellect) and *indriyātha* (sensorial processing:output) are in harmony, leading to a wholesome mental health. Any disturbance in any one or more than one of the axis leads to a wholesome mental ill-health.

Carka in his treatise **Charaka Samhitā [12]** in the section *Nidānsthānam* (Section on Diagnosis—Chapter VII) has considered mental ill-health as essentially a result of a disequilibrium brought about by the unwholesome interaction between the individual and their surrounding environment. This interaction operates through an axis consisting of three fundamental factors, viz. *kāla* (time rhythm), *būddhi* (intellect) and *indriyātha* (sensorial inputs). Time rhythm is considered to be the interaction of an individual with the daily experience of life. These experiences can vary from being pleasant to extremely unpleasant. The response of the individual to these events must be objective and dispassionate. Any disequilibrium in this axis leads to wide-ranging altered behavior (Figure 1). The common mental ailments classified by Charaka are *unmāda* (psychosis), *apāsmara* (convulsive disorders), *cittodvegā* (anxiety disorders), *cittavasada* (depressive illness), *mada* (alcoholism and drug abuse), *sanyāsa* (complete indifference to the environment, surroundings and world) and *mūrchā* (loss of sensorial inputs, resulting in coma). Besides their aetiopathogenesis, Carak has also described signs, symptoms and behavioral alterations in different psychiatric disorders and classified them accordingly, which we will consider later.

## 2. Āyurvedic Concept of Life Span, Health and Brain Aging

### 2.1. Life Span

*Āyurveda*, like any other Indian tradition, considered the total life span of an individual to be over one hundred years. The ***Rig Veda***, first of the four Vedas, considered to be the earliest recorded book of wisdom in human civilization, wishes every individual to lead a healthy life of a hundred years: “*jeevem sárdah śatam*”. In the earlier phase of *Āyurveda*, the total life span was divided into the following three categories: *bālyavasthā* (childhood): 24 years; *yuvāvasthā* (young): 44 years and *vŗidhavasthā* (old age): 48 years. Thus, total life span was considered to be 116 years. However, later Sūsŕuta [13] gave an elaborate and systematic classification of age as follow:

*Balyavāyya* (childhood): 0–16 years. The *balyavāya* further constituted three stages of *kśirpa* (milkfed), *kśirannada* (weanlings) and *annadā* (fed on cereals). 

The next stage of life, viz. *madhya vāya* (middle age), spanned from 16 to 70 years and consisted of four stages, *vŕdhi* (period of growth or evolution): 16–20 years; *yūvāna* (youth): 21–30 years; *sampūrna* (when the growth is complete): 31–40 years and *hāni* (period of involution or gradual decline): 41–70 years. 

The last stage life was *vŕidha vāya* (old age), which lasted from 71 to 100 years.

### 2.2. Health

The conceptual frameworks of health in *Āyurveda* are essentially based upon the doctrine operating through the principle of *sāmānya* and *viśeśa*, *i.e.*, Heterology *versus* Homology. This was further extrapolated by Sūśruta as *loka-puruśa sāmya* (equilibrium between an individual-microcosm and cosmos-macrocosm) by establishing the continuum between the ecological triangle of *soma-sūrya-anila* (moon-sun-air) with the *tridośika* (three-dimensional) bio-triangle of *kapha-pitta-vāta*. Here it would be pertinent to emphasize that *kapha*, *pitta* and *vata* cannot be translated into one word, as many western scholars and educated Indians tend to do and translate *kapha* as phlegm, *pitta* as bile and *vata* as wind, thereby giving a most erroneous interpretation. *Kapha* can be appropriately translated as one of the body humors, which forms the solid substratum of the body, including immune strength; *pitta* is another bio-humor, which is responsible for the entire digestive and metabolic functions; and *vāta* is the bio-humor responsible for energetic and neural activities [14].

As an ecological balance between *soma-sūrya-anila* (moon-sun-air) sustains the entire universe, the physiological homeostasis (constancy of milieu interior) of the human body is sustained by a harmonious balance between principal bio-humors sustaining the immunity, metabolic, energetic and neural activities. The individual living being is considered a miniature replica of the universe and comparatively similar activities are taking place inside the microcosm of the human body as are occurring universally in the macrocosm.

Suśruta has defined in Chapter XXV of *Sūtrasthanam svāsthya*, *i.e.*, total-health, in the following words: “A healthy person is he whose humors and metabolic state are in equilibrium, whose functional activities of the tissues and excretory products (*i.e.*, the physical state) are in balance, and the soul, senses and mind (*i.e.*, the mental state of the body) feel well” [13]. This comprehensive definition describes both the healthy features of the physical body and physical wellbeing, as well as the states of the sensorial, mental and spiritual qualities of wellbeing. Thus, health was considered as a state of physical, mental, spiritual and sensorial/social wellbeing; a similar definition was later adopted by WHO as “health is a state of physical, mental, social and spiritual well being”.

Taking the Suśrutu definition of health further, the founding fathers of independent India under the Chairmanship of the first Prime Minister Pandit Jawaharlal Nehru had defined health as “a positive state of well-being in which harmonious development of physical and mental capacities of the individual leads to the enjoyment of a rich and full life. It is not a negative state of mere absence of disease. Health further implies complete adjustment of the individual to his environment, physical and social.” [15].

### 2.3. Brain Aging

Aging is a generalized systematic involution of a living body, its organs, tissues and cells. The brain is considered to be the most vulnerable organ to this aging process, as neurons lack a robust regenerative capacity. Both the great exponents of *Āyurveda*, viz. Caraka and Śusrūtu, have propounded that *medha*, *i.e.*, the core cognitive function of brain starts declining from the fourth decade of life onwards, and after the eighth decade of life, the loss of *būddhi* or decision making capacity becomes imperative, leading to senile dementia. Therefore, in order to help fulfill the cherished desire of every human being to live the optimum 100 years of life-span with healthy functioning brain and senses, *rasāyanā* rejuvenating therapies were introduced.

Aging is associated with various degenerative changes like functional hormone deficiency state and accumulation of oxidative damage to DNA, proteins and lipids, which results in interference with normal function and produces a decrease in stress responses. Since the inception of civilization, elderly persons have been predominantly more prone to age-related brain degenerative disorders than actual gross somatic aging.

Hence, Āyurveda introduced a unique approach to diet and nutrients and a *rasāyanā* rejuvenating therapy to decelerate aging and manage geriatric problems [16].

Though the use of *rasāyanā* was primarily to impart longevity and promote a healthy prolonged life-span, the primary attribute of *rasāyanā* is nutrition, *i.e.*, overall nourishment of body. This primary effect of nourishing the body results in longevity and decelerating the process of aging. Hence, it has been advocated that *rasāyanā* should be used throughout life, especially in early and middle ages [17].

Different Āyurvedic texts have prescribed selected *rasāyāna* suitable for different age groups. By using this approach, a physician can prevent or minimize specific age-related losses. It has been suggested that an individual loses one important physiological attribute of life every decade after the completion of childhood. The fourth and ninth decades of human life have been considered to be crucial for brain-aging. The *rasāyanās* are thus selected for respective decades: (a) promote, prolong or maintain a desired effect, especially in the early decades of life and (b) prevent loss in the later decades of life (Table 1).

**Table 1:** Different *rasāyanās* to be taken in different decades of life [18].

Decade of Life	Desired Effect/ *Loss of Physiological Attributes*	*Rasayana* Plants
1–10	Formation, development and promotion of speech *childhood/boyishness*	*Vāca* (*Acorus calamus*; family: Acoraceae).
11–20	Growth; attaining puberty and a healthy growth of brain and body.	*Brahmi* (*Bacopa monniera*; family: Scrophulariaceae); Bala (*Sida cordifolia*; family: Malvaceae); and *Kasmari* (*Abutilon indicum*; family: Malvaceae).
21–30	Promotion of intellect/*luster/complexion*	*Brahmi* (*Bacopa monniera*; family: Scrophulariacae) and *Amalaki* (*Emblica officinalis*; family: Euphorbiaceae).
31–40	*Cognitive functions and memory*	*Brahmi* (*Bacopa monniera*; family: Scrophulariacae) and *Shankhpushpi* (*Evolvulus alsinoides*; family: Convolvulaceae.
41–50	*Skin*	*Brahmi* (*Bacopa monniera*; family: Scrophulariacae) and J*yotishmati* (*Celastrus peniculatis*; family: Celastraceae).
51–60	*Vision*	*Brahmi* (*Bacopa monniera*; family: Scrophulariacae) and *Jyotishmati* (*Celastrus peniculatis*; family: Celastraceae).
61–70	*Virility and procreative prowess*	*Ashwagandha* (*Withania somnifera*; family: Solanaceae) and *Brahmi* (*Bacopa monniera*; family: Scophulariacae).
71–80	*Physical strength, sensorial and muscular coordination*	*Amalaki* (*Emblica officinalis*; family: Euphorbiacae) and *Brahmi* (*Bacopa monniera*; family: Scrophulariacae).
81–90	*Discriminative faculty, intelligence and decision making capacity*	*Brahmi* (*Bacopa monniera*; family: Scrophulariacae).
91–100	*Mental coordination, locomotor action and mobility*	*Brahmi* (*Bacopa monniera*; family: Scrophulariacae).

## 3. Diet and Nutrition

### 3.1. Diet and Health in Carak-Samhitā

Chapter V of the *Sūtrasthānam* (Fundamentals) of **Carak-Samhitā** [12] is specifically devoted to the quantity of diet and *dincaryā* (daily routine) and Chapter VI to the quality of diet and *ŕitucaryā* (mode of living in different seasons). Caraka had emphasized that following the *dincaryā* and *ŕitucaryā* regimens, one can ensure good physical and mental health and longevity.

The quantity of food, dependent upon the power of digestion and the quantity of food that gets digested in time without disturbing normalcy, should be considered as the measure of proper quantity. The food taken in proper quantity definitely provides strength, complexion and a happy life to a person without vitiating his normal physiological functions.

For healthy living, Caraka has advised that a wise person should meticulously follow the duties relating to his own body in the same way as a civic authority is conscientious of his duties to the city and a charioteer to those of the chariot.

Caraka had not prescribed any universal diet, but advocated that an individual should be the best judge of his own diet and should eat only that food the leads to the promotion of physical health, mental prowess and complexion according to different seasons.

### 3.2. Diet and Health in Suśrūta-Samhitā

Like Caraka, Suśrūta has also laid emphasis on *ŕitucaryā* (seasonal regimen). Chapter 6 of Sūtra-Sthānam (Fundamentals, Plastic-Surgery & Pharmaceutical Considerations) is devoted to *ŕitucaryā*.

The lunar year of *Āyurveda*, consisting of twelve months, was sub-divided into two solstices, viz. southern and northern. Out of the two, the southern solstice includes the rainy, autumn and early winter seasons. During these seasons, the moon has ascendancy, as the sun is further away from the axis of the earth. As a result, the tastes get concentrated in the plants of both medicinal and food value, and therefore, every living being progressively becomes stronger. In contrast, during the northern solstices, which include winter, spring and summer, there is ascendency of the sun and, hence, bitter, astringent and pungent tastes become prominent; this results in the depletion of medicinal and nutrition values of plants and leads to a gradual decline in the strength of every living being.

The food partaken should be such that it gives rise to exhilaration, physical strength and vigor, mental competence, nourishment, energy, satisfaction and pleasure. The food taken in proper amounts is digested easily and maintains the equilibrium of *dhātus* (principal body tissues) and *dośas* (humors or biofactors controlling body physiology).

**Figure 2:**
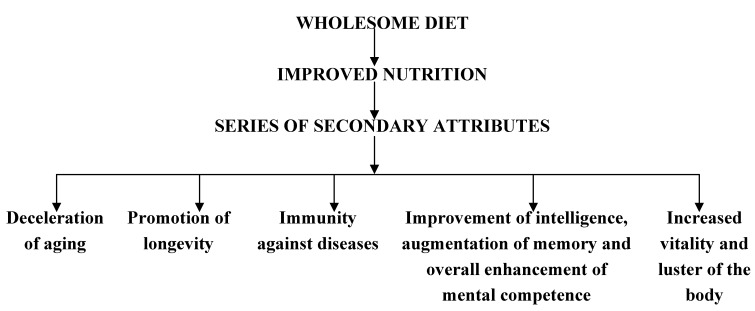
*Āyurveda* advocates the regular use of a wholesome diet, which will help an individual to achieve his cherished goal of leading a healthy life of 100 years.

Thus, *Āyurveda*, lays a great deal of emphasis on leading a healthy life by following *dincaryā*, *ŕitūcaryā* and partaking of a nutritious and wholesome diet. Such a regimen helps an individual to fulfill his cherished goal of leading a healthy life of 100 years (Figure 2).

### 3.3. Bhūtvidyā (Psychology, including Clinical Psychology and Psychiatry)

**Figure 3:**
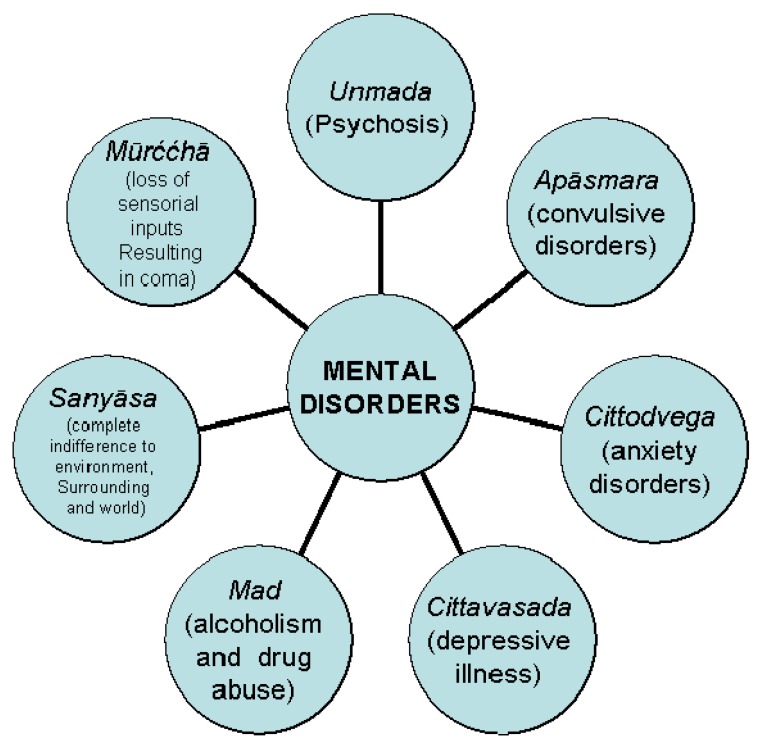
*Āyurvedic* forms of mental disorder.

**Figure 4:**
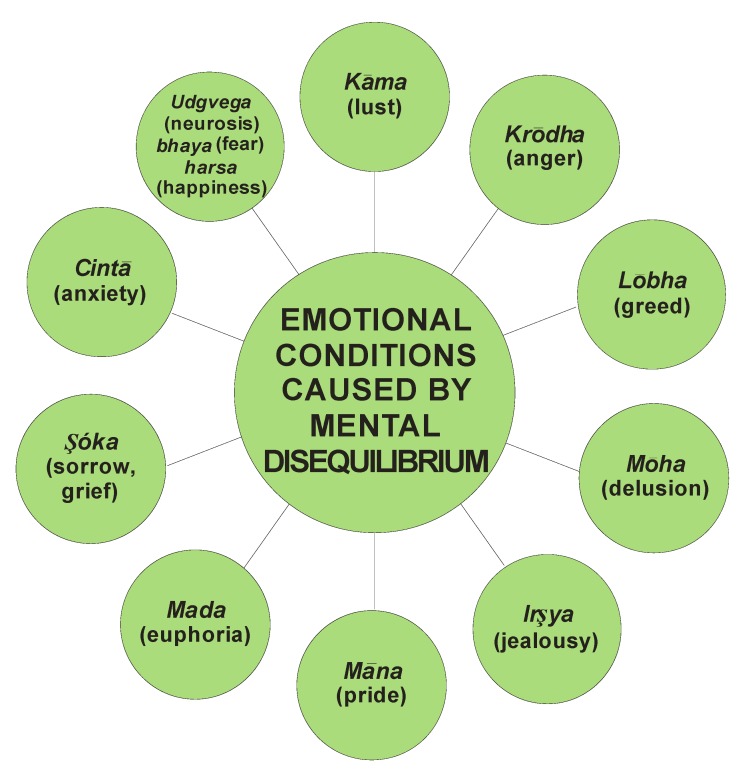
The various emotional conditions caused by mental disequilibrium in *Āyurveda*.

**Figure 5:**
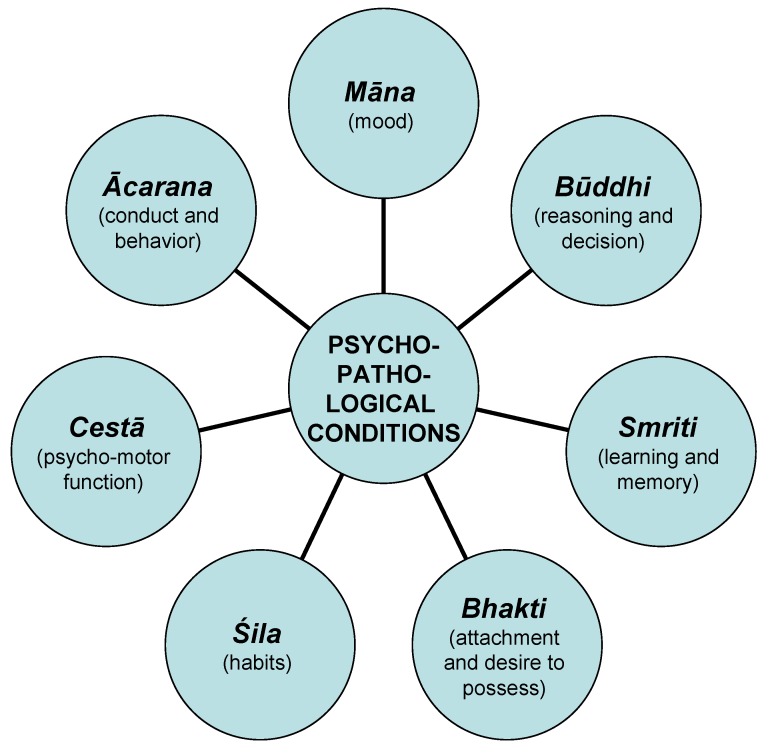
Psychopathological conditions induced by various psychological factors.

**Figure 6:**
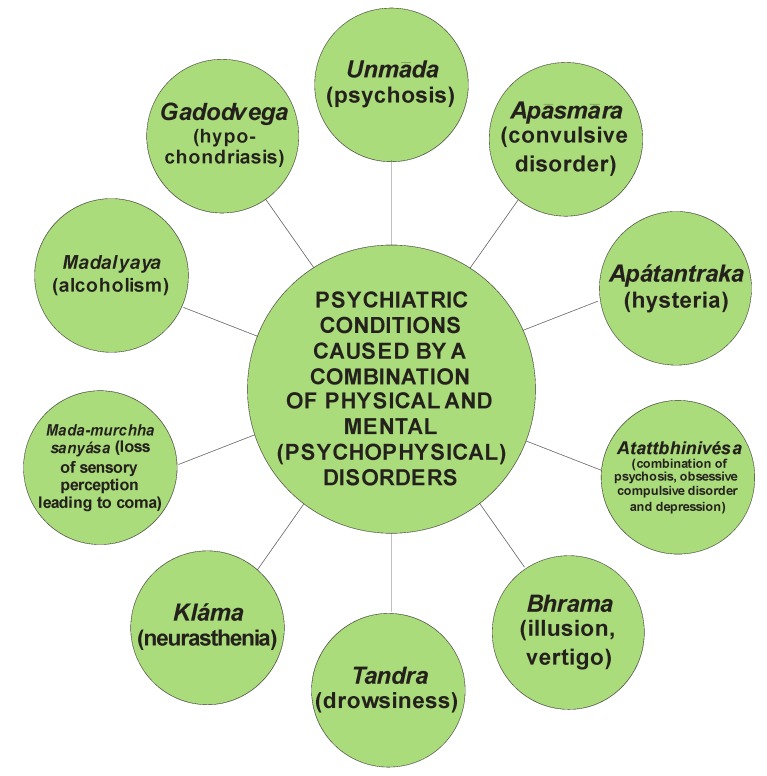
The various psychiatric conditions caused by a combination of physical and mental (psychophysical) disorders.

*Bhūtvidyā* was an important, advanced and evolved branch of *Aśtānga-Āyurveda*. It had described mental (Figure 3), psychiatric (Figure 4) and psychological (Figure 5) disorders caused purely by mental disorders and a combination of physical and mental disorders (Figure 6).

## 4. *Rasayana* and Treatment of Psychiatric Disorder

The various drugs used for the treatment of mental health are classified as *medhya rasāyanās*. These act as brain tonics and adaptogens and are specific molecular nutrients for the brain promoting health, thus leading to the alleviation of various behavioral disorders, including memory impairment. The *medhya rasāyanās* afford biological nourishment of the brain, producing tranquility of mind, mental concentration and promotion of memory.

### 4.1. The Rasayana Concept

**Figure 7:**
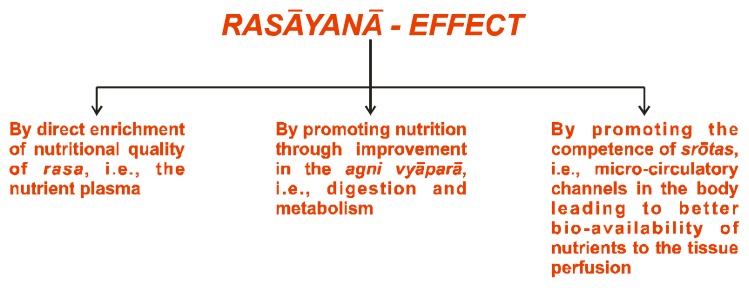
The three-dimensional effect of *rasāyanā*.

**Figure 8:**
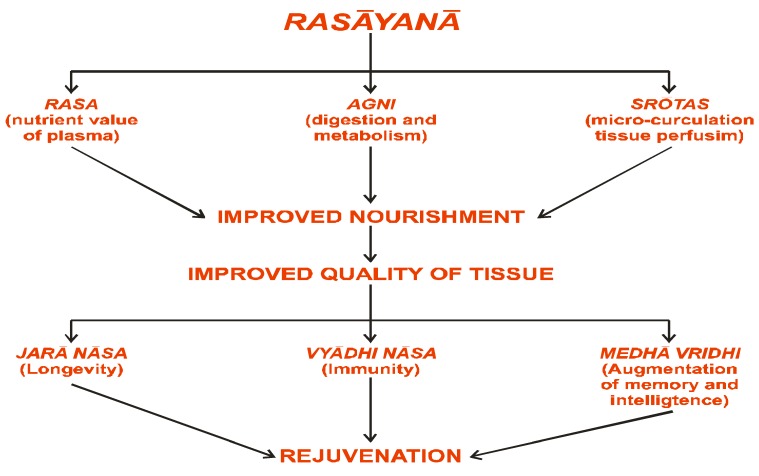
The rejuvenation channel of *Rasāyanā* effect.

In *Āyurveda*, *rasāyanā* (or science of rejuvenation) is a unique concept and is one of the eight specialized branches of *Aśtāng-Āyurveda*. The main object of *rasāyanā* therapy is the management of age-related disorders. The principal physiological effect of *rasāyanā* is to improve and revitalize the physiological and endocrine functions of the body, to decelerate the aging process and to make an individual more responsive and resistant to disease, *i.e.*, to improve body function by strengthening the immune system (Figure 7, Figure 8). The *rasāyanās* are postulated to act through a psycho-endocrinological immune axis [17].

### 4.2. Ācara-Rasāyanā (Rejuvenating Code of Conduct)

**Figure 9:**
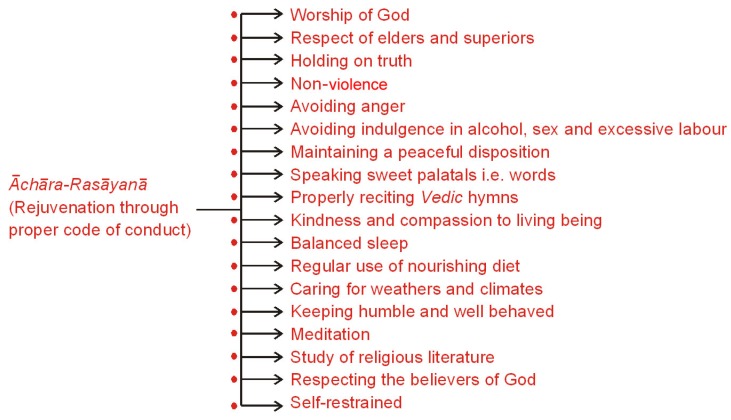
An aspirant who lives such a life and practices *sadāchār* (good conduct) achieves the *rasāyanā* effect, immunity, longevity and intellectual power. He is free from emotional disturbances, is less stressful, has pronounced anabolic static and, thus, leads a happy and healthy life.

It is pertinent to emphasize that besides a nutritious diet, practice of *āchāra*-*rasāyanā* (rejuvenation through proper code of conduct) was also strictly advocated (Figure 9).

In essence, *Rasāyanā*, or science of rejuvenation, deals primarily with geriatric problems and lays great emphasis on specific measures to improve the physical, physiological and psychological adaptation to various pathological changes related to the problems of aging. It considers aging as a gradual decline in the adaptive behavior of an individual to environmental changes. Several scientific studies conducted to evaluate the effects of *ācāra rasāyanā*, diet and treatment have revealed clinical improvements and significant positive alterations in specific biochemical parameters, like plasma cortisol, catechol and indole amines, *etc.* The EEG studies have revealed accentuation of alpha and beta waves and diminution in spikes of delta waves. It provides significant improvement and correction in the physiological and endocrine functions of the body and makes an individual more responsive and responsive to various geriatric diseases by augmenting body immunity [19,20,21,22,23]. 

**Figure 10:**
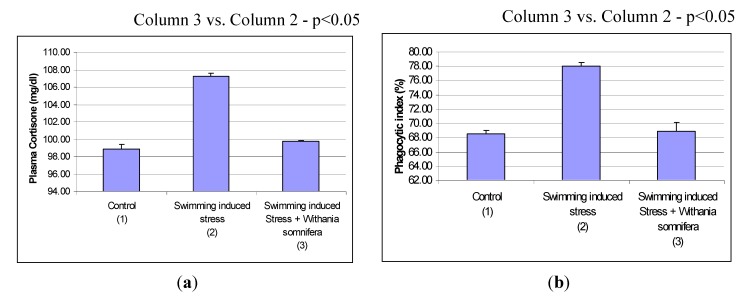
Effects of *Withania somnifera* on stress-induced plasma corticosteron (**a**) and phagocytic index (**b**).

Archana and Namashiyam [24] have shown significant alterations in humoral basis in control and swimming-induced stressed rats when treated with the roots of *Withania somnifera* (Figure 10a,b).

**Figure 11:**
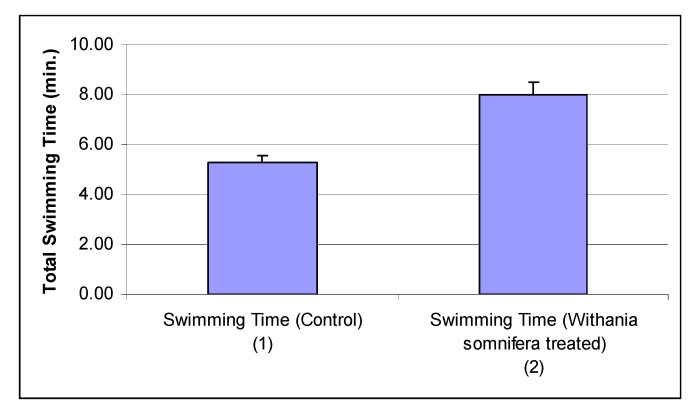
Effects of *Withania somnifera* on swimming endurance time. Column 2 *vs.* Column 1—*p* < 0.05.

*Withania somnifera* also significantly enhanced the endurance performance as measured by total swimming time (Figure 11).

## 5. *Medhya-Rasayana* (Memory Enhancing and Rejuvenating) Plants

There are various classifications of *rasáyanás*, *i.e.*, according to the mode of administration (Carak) or site of action (Sūśrutu), *etc.* Here, we are concerned with *medhyā rasāyanā* used for rejuvenation of brain and mental health and to promote intellect and memory. The names of plants used for treatment of mental disorders have varied in various texts and have been summarized in an authoritative contemporary text [22] in Table 2.

**Table 2:** A list of plants used in *Āyurvedic* medical practice to treat mental disorders.

Sl. No.	Botanical Nomenclature	*Āyurvedic* Nomenclature	Family
1	*Acorus calamus*	*Vacha*	Aracceae
2	*Alternanthera sessilis*	*Matsyakshi*	Amarnanthacceae
3	*Argyreia speciosa*	*Vidhara*	Convolvulaceae
4	*Asparagus racemosus*	*Shatavari*	Liliaceae
5	*Bacopa monniera*	*Brahmi*	Scrophulariaceae
6	*Celastrus paniculatus*	*Jyotishmati*	Celastraceae
7	*Centella asiatica*	*Mandukparni*	Umbelliferae
8	*Clerodendrum infortunatum*	*Bhant*	Verbanaceae
9	*Clitoria ternatea*	*Aparajita*	Leguminosae
10	*Convolulus pluricaulis*	*Shankhpushpi*	Convoluvalaceae
11	*Curculigo orchiodes*	*Krishna musli*	Amaryllidaceae
12	*Dioscorea bulbifera*	*Varahi kand*	Dioscoreacae
13	*Enhydra fluctuans*	*Hilmochika*	Compositae
14	*Glycyrrhiza glabara*	*Yashtimadhu*	Leguminosae
15	*Jasminum sambac*	*Bela*	Oleaceae
16	*Mucuna pruriens*	*Kewanch*	Leguminosae
17	*Nardostachys jatamansi*	*Jatamansi*	Valerianceae
18	*Piper longum*	*Pippali*	Piperaceae
19	*Terminalia chebula*	*Haritaki*	Combretaceae
20	*Tinospora cordifolia*	*Amrita*	Menispermaceae
21	*Valeriana wallichii*	*Tagar*	Valerianaceae
22	*Vitex negundo*	*Nirgundi*	Verbenaceae
23	*Withania somnifera*	*Ashwagandha*	Solanaceae

Thus, we find that there have been several plants traditionally used for thousands of years as *rasāyanā* in *Āyurveda*. Several of them have a very interesting antioxidant profile of activities [25]. Some authors have postulated that because of the pronounced anti-oxidant activity, *rasāyanā* plants would be effective in treating several reactive oxygen species mediated disorders, like memory impairment, senile dementia of the Alzheimer’s type and Parkinson’s disease [26].

### Bacopa monniera

**Figure 12:**
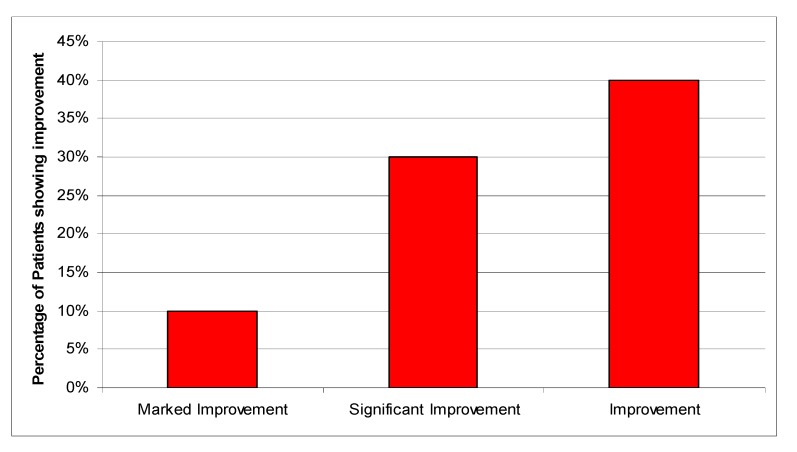
Overall effect of bacopa treatment [28].

We shall illustrate the unique properties of the *rasāyanā* plants by taking the example of *Bacopa monniera*. It is an extremely important plant of *Āyurveda* used since the time of Rig-Veda (*c*. 3500 BCE), and its name is derived from Lord Brahmā, the creator of universe in Hindu mythology and believed to be the originator of *Āyurveda* [27]. As has been shown in Table 1, Bacopa has been recommended in various *Āyurvedic* texts to be taken during each decade of life after the first decade. It has been classified as *medhya-rasāyanā* (memory enhancing and rejuvenating), as well as *aindra-rasāyanā* (increasing longevity and promoting progeny). Because of its traditional importance and unique traditional therapeutic claims of alleviating old age and age-related diseases, promoting memory and intellect, enhancing life-span, providing nourishment, excellence, clarity of voice, complexion and luster and being efficacious in a wide variety of psychiatric disorders, like hallucination, schizophrenia, obsessive compulsive disorder, severe psychosis and also producing a photographic memory, it has been investigated in different laboratories in India, particularly in the Central Drug Research Institute (CDRI), Lucknow. We will take the *medhya-rasayana* properties later. The *aindra-rasāyanā* effect has been negligibly investigated, except perhaps for a M.D. (*Āyurveda*) thesis by Diggavi [28]. The methodology used in the thesis is not very sound, chiefly because an unstandardized bacopa powder was administered to 10 patients randomly selected from the OPD of the *Vājikarana* (Sexology) section of the *Kayāchikitsa* department of Integrated Post-Graduate Teaching and Research in *Āyurveda* (IPGTRA) of Gujarat *Āyurveda* University, Jamnagar. All the patients were fulfilling the clinical *Āyurvedic* criteria of diagnosis of *Klaibyā* (Male Sexual Dysfunction). The total course of treatment was 30 days with 5g of bacopa powder three times a day with milk. The study was open-ended. Although increase in several parameters, including semen parameters, were observed, these were not significant. However, marked increase was observed with bacopa treatment on the sexual health in several parameters on an arbitrarily chosen subjective scale. Overall, bacopa treatment showed a varying degree of improvement in the volunteers (Figure 12).

This aspects merits further investigation before the traditional claim of bacopa being an *aindra-rasāyanā* can be accepted scientifically.

However, because of the investigations done at CDRI, the traditional claim of bacopa being *medhya-rasāyanā* has been scientifically accepted.

A systematic chemical investigation of the plant by Basu *et al.* [29,30] and Chatterjee *et al.* [31] revealed that Bacopa contains the following constituents: Bacoside A (64.28%), Bacoside B (27.11%), betullic acid (4.58%), D-mannitol (0.83%), stigmasterol (0.54%), β-sitosterol (0.58%) and stigmastanol (2.08%). The activity of the ethanolic extract was traced to the mixture of the triterpenoid saponins designated as Bacosides A and B. Bacoside A is levo-rotatory and Bacoside B is dextro-rotatory.

Bacoside A was the major component of the plant and comprised two sets of saponins. One set was derived from pseudojujubogenin, which upon acid hydrolysis furnished four triterpenoid transformation products, viz. Bacogenins A_1_, A_2_, A_3_ and A_4_ [32]. The second set of saponins was derived from jujubogenin, which upon acid hydrolysis yielded two triterpenoids with triene side chains as transformation products. These triterpenoids were designated as Bacogenins A_4_ (*trans*) and (*cis*). A standardized extract of bacopa containing a minimum of 55% ± 5% of bacosides with an optimum concentration of Bacogenins (especially Bacogenin A_4_), *vis-a-vis* memory enhancing effect, was developed. This was termed as bacosides-enriched standardized extract of bacopa (BESEB CDRI-08).

To begin with, the BESEB CDRI-08 was tested in a critically selected learning model, which would produce well-distributed and extensive cellular changes during learning discrimination and involve a reversal of innate behavior. For instance, rodents being nocturnal animals prefer darkness in preference to light. If the learning model is such that an entry of the experimental animal in the lighted alley of the training maze escapes punishment and an entry in the dark alley is punished, then the model would serve the purpose of reversing the innate behavior of a preference of darkness. The model thus chosen was a foot-shock motivated brightness discrimination task on rats in a semi-automatic Y-maze described in detail by [33,34,35].

It is also important to use a battery of diversified tests whenever attempting to measure the effect of any drug on the level of motivation or emotion [36]. Hence, the memory enhancing effects of BESEB CDRI-08 were evaluated in a battery of tests consisting of positive (reward), as well as negative (punishment), reinforcements, on the labile phase of memory (when the memory is in formative stage) and stable phase of memory (when the memory formation has taken place). The training methods should also allow a clear demarcation of the three memory phases, viz. acquisition, consolidation and retention, and also clearly distinguish between the successive stages of short-term, intermediate and long-term memories. The foot-shock motivated brightness discrimination reaction fulfilled most of these criteria in as far as the training was completed in one session to produce a labile memory formation and it was with a negative reinforcement (punishment). However, to obtain predictive and confirmative evidence, the BESEB CDRI-08 was tested in other learning models of active avoidance response and Sidman’s continuous avoidance response (to produce a stable phase of memory with negative reinforcement in multi-session or interval sessions) and conditioned taste aversion response (to produce a labile phase of memory with positive reinforcement).

The result of these investigations confirmed that BESEB CDRI-08 has a significant facilitatory effect on all the forms of memory, *i.e.*, short, intermediate and long-term memories in all the three phases, viz. acquisition, consolidation and retention [37,38,39,40,41,42,43,44].

Recent studies have shown that BESEB CCDRI-08 influenced the serotonergic system by elevating the level of 5-HT and up-regulating the expression of 5-HT_3_A receptor, possibly by interacting with the cholinergic system [38]. The improvement observed in the hippocampus-dependent learning model was possibly due to the combined effect of serotonergic and cholinergic systems. This is in conformity with other findings that multiple neurotransmitters are involved in the learning memory process [39,40,41,42,45,46,47,48,49].

**Figure 13:**
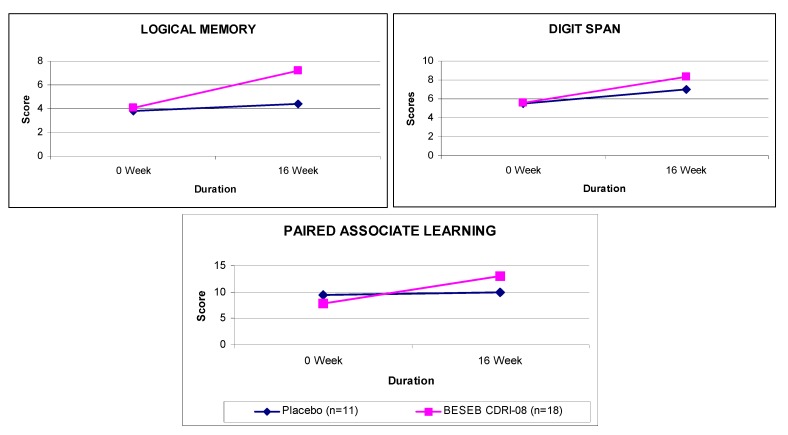
Effect of BESEB CDRI-08 (50 mg × 2 daily) on children suffering from attention deficit hyperactivity disorder.

**Figure 14:**
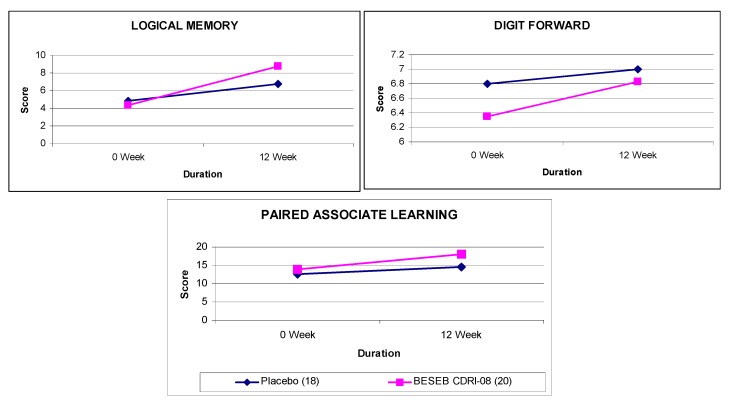
Effect of BESEB CDRI-08 (150 mg × 2 daily) on elderly subjects suffering from age associated memory impairment.

BESEB CDRI-08 was found to be safe in regulatory sub-acute toxicity [50] and teratogenicity studies [51]. No abnormalities were observed in genotoxicity and mutagenicity [52]. The product was efficacious in the treatment of children suffering from attention deficit hyperactive disorder [53] and adults suffering from age-associated memory impairment [54,55,56] (Figure 13, Figure 14).

BESEB CDRI-08 has shown its significant effectiveness in improving the cognitive symptoms associated with inattention, impulsiveness in children suffering from Attention Deficit Hyperactivity Disorder [53] and also showed significant improvement in several memory test scores on elderly subjects suffering from Age Associated Memory Impairment [54,55,56]. The extract has thus shown its putative potentials in the treatment of cognitive impairment. Considering its other interesting profile of activities, like anti-anxiety [17], the extract appears to be a potential treatment of the total loss of memory. Blood analysis would also be an important avenue for research on this extract, especially the identification of antioxidant markers.

## 6. Conclusion

*Āyurveda* in essence relies substantially on nature, and it is only to provide assistance to nature that drug and other therapeutic measures are applied. *Āyurveda* also relies on *swabhāvoparma* (recession by nature) and dietics, and drug regimens are based on this principle. The therapeutic approach is based on *samśodhana* (preventive and curative) measures by augmenting the natural resistance of the patient. Besides the unique branch of *rasāyanā*, due emphasis has also been laid down on the code of good conduct to lead a healthy and long life. Diseases can be prevented by following a proper daily routine and seasonal living. The rich repository of unique efficacious plants mentioned in *Āyurveda* has elicited a significant hope in current drug discovery research, which is characterized by a target-rich and lead-poor scenario [55,56]. Increased interest is being witnessed at a global scale to evolve drug discovery strategies based on natural products and traditional medicine, and it is being increasingly realized that development of new drugs need not always be restricted to new molecular entities [57].

In recent years, health planners and practitioners are recognizing the value of traditional medical systems like Āyurveda [58]. The medicinal plants from indigenous pharmacopoeias like the compendia of Caraka and Suśrūta have shown significant healing power and have been postulated to be effective in a wide variety of diseases, ranging from allergic rhinitis [59], cancer [60] to Alzheimer’s disease [61].

As a result of a global interest of vast dimension in herbal products, many plant-based products under the guise of food supplements have entered the markets of the USA and other Western countries. These have been termed nutraceuticals containing many plants lacking in botanical verification, besides evidence for efficacy or safety. This problem is further compounded by the fact at present that there is no globally accepted regulatory guidelines to ensure that these herbal products are what they claim, are as efficacious as claimed and, most importantly, how safe they are.

The bacopa extract designated BESEB CDRI-08 is derived from an important *rasāyanā* plant, which has been in use in the Indian system of medicine sine 3000 BCE. It is a standardized extract of the authentic identified plant, found to be efficacious in improving cognitive functions in a wide variety of test situations; the standardization parameters are well defined and regulatory toxicity studies have shown it to be safe.

The Āyurvedic concept of diet and good conduct provides another traditional basis of good health and it would be worthwhile to do some research in this direction. Several studies have shown that practicing a good conduct of behavior combined with a nutritious diet and treatment with *rasāyanā* plants lead to a highly significant improvement in combating various geriatric diseases and augmenting body immunity [19,20,21,22,23].

Āyurvedic texts have described in vivid detail a very wide array of ingredients, including a proper moral code of conduct and personal hygiene, to lead a long, healthy life with enhanced brain functions.
